# Systematic pan-cancer landscape identifies CARM1 as a potential prognostic and immunological biomarker

**DOI:** 10.1186/s12863-021-01022-w

**Published:** 2022-01-16

**Authors:** Yingqi Qiu, Hao Wang, Peiyun Liao, Binyan Xu, Rong Hu, Yulu Yang, Yuhua Li

**Affiliations:** 1 Department of Hematology, Zhujiang Hospital, Southern Medical University, No. 253 GongyeDadaoZhong, Guangzhou, Guangdong 510280 People’s Republic of China; 2 Bioland Laboratory (Guangzhou Regenerative Medicine and Health Guangdong Laboratory), Guangzhou, Guangdong 510005 People’s Republic of China

**Keywords:** CARM1, Pan-cancer analysis, Prognosis, Immune, Tumor microenvironment

## Abstract

**Background:**

Belonging to the protein arginine methyltransferase (PRMT) family, the enzyme encoded by coactivator associated arginine methyltransferase 1 (CARM1) catalyzes the methylation of protein arginine residues, especially acts on histones and other chromatin related proteins, which is essential in regulating gene expression. Beyond its well-established involvement in the regulation of transcription, recent studies have revealed a novel role of CARM1 in tumorigenesis and development, but there is still a lack of systematic understanding of CARM1 in human cancers. An integrated analysis of CARM1 in pan-cancer may contribute to further explore its prognostic value and potential immunological function in tumor therapy.

**Results:**

Based on systematic analysis of data in multiple databases, we firstly verified that CARM1 is highly expressed in most tumors compared with corresponding normal tissues, and is bound up with poor prognosis in some tumors. Subsequently, relevance between CARM1 expression level and tumor immune microenvironment is analyzed from the perspectives of tumor mutation burden, microsatellite instability, mismatch repair genes, methyltransferases genes, immune checkpoint genes and immune cells infiltration, indicating a potential relationship between CARM1 expression and tumor microenvironment. A gene enrichment analysis followed shortly, which implied that the role of CARM1 in tumor pathogenesis may be related to transcriptional imbalance and viral carcinogenesis.

**Conclusions:**

Our first comprehensive bioinformatics analysis provides a broad molecular perspective on the role of CARM1 in various tumors, highlights its value in clinical prognosis and potential association with tumor immune microenvironment, which may furnish an immune based antitumor strategy to provide a reference for more accurate and personalized immunotherapy in the future.

**Supplementary Information:**

The online version contains supplementary material available at 10.1186/s12863-021-01022-w.

## Background

Due to the heterogeneity and diversity of tumors, the deficiency of effective biomarkers represents one of the main bottlenecks restricting the development of cancer medicine. The accumulated big data analysis of any gene of interest has become a powerful means to explore the complex process of tumorigenesis and development. Analyzing gene expression and related genetic modification allows us to evaluate its clinical prognosis and explore related signaling pathways, which could help to find new immunotherapy targets.

**Fig. 1:**
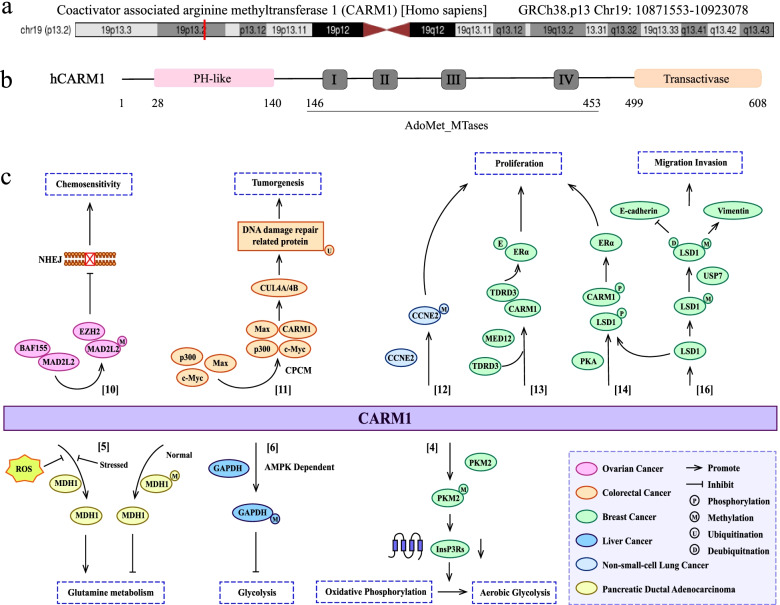
Basic information about CARM1. **a** Genomic location of human CARM1. **b** Protein structure diagram of human CARM1. **c** According to the reported studies, the carcinogenic pathways CARM1 was involved in across different cancers are shown graphically. The related references are also indicated

CARM1, also known as PRMT4, located in Chr19p13.2 (Fig. 1a), was first identified as an arginine methyltransferase that introduces asymmetric methylation of arginine residues in histone H3 and other chromatin-associated proteins [1]. With regard to human CARM1 protein, it is composed of an N-terminal pleckstrin homology-like domain (PH-like), a C-terminal transactivase domain, and a central catalytic domain containing the four conserved PRMT motifs (Fig. 1b). The N- and C-terminal domains of CARM1 are vital for substrate recognition and transcription-mediated activation [2], and the motifs in central catalytic domain are essential for binding of the cofactor S-adenosyl methionine (SAM) and the substrate arginine [3]. Long known as a transcriptional coactivator, recent studies have shown that it is also involved in the regulation of metabolism [4–6], autophagy [7], RNA regulation [8] and early mammalian development [9]. Recently, accumulating evidence has suggested that CARM1 also has an impact on the occurrence and development of tumors [10–14]. Existing studies on exploring the mechanisms of CARM1 methylation affecting tumor progression have shown that CARM1 is a coactivator of several cancer-related transcription factors and can be involved in promoting tumor cell proliferation and metastasis by methylating cancer-related transcription factors, including NF-κB, p53, steroid receptors and so on, and its high expression is associated with poor prognosis of tumors [15]. For example, in the most studied breast cancer, CARM1 could methylate the R838 site of lysine demethylase 1 (LSD1) to promote the binding of deubiquitinase USP7, resulting in the ubiquitination and stabilization of LSD1, thereby promoting the invasion and metastasis of breast cancer cells [16]. In addition, CARM1 has been found to be involved in regulating metabolic pathways in tumors. Metabolic reprogramming is a hallmark of cancer. In breast cancer cells, methylation of the key glycolytic enzyme pyruvate kinase M2 isoform (PKM2) by CARM1 shifts the metabolic balance from oxidative phosphorylation to aerobic glycolysis, producing a large amount of ATP, so as to promote tumor cell proliferation and migration [4]. Nevertheless, CARM1 is up-regulated when glucose starvation, followed by methylation of GAPDH to inhibiting glycolysis, thereby suppressing tumor cell proliferation in liver cancer cells [6], which is to some extent consistent with the results of the correlation analysis between CARM1 expression and liver cancer prognosis described in our work. Current evidence about effects of CARM1 on various cancers has been shown in Fig. 1c and Table 4 [5, 6, 13, 16–25].

However, researches on CARM1 in cancers are only started in recent years, and limited to several kinds of tumors. There is still no systematic pan-cancer evidence about the relationship between CARM1 and multiple tumor types based on big clinical data. Our work, for the first time, used multiple databases containing The Cancer Genome Atlas (TCGA) project, cBioPortal, Human Protein Altas (HPA) and so on to conduct a comprehensive pan-cancer analysis of CARM1. A group of factors, such as gene expression, survival status, genetic alteration, tumor mutation burden (TMB), microsatellite instability (MSI), methyltransferases genes, immune infiltration, and relevant cellular pathway, are included to investigate the potential associations between CARM1 and the pathogenesis and clinical prognosis of different cancers, providing a basis for further understanding the role of CARM1 in tumor immunotherapy.

## Results

### Expression profile of CARM1 across normal tissues and cancer samples

In this work, we aimed to investigate the role of human CARM1 in tumorigenesis and development. As mentioned above, CARM1 protein is usually composed of a N-terminal pH like domain (cl17171), a C-terminal transactivase domain, and a central catalytic domain (cl17173), and its structure is conserved among most species (Fig. S1a, see Additional file 1, e.g., *H. sapiens*, *M. mulatta*, *R. norvegicus*, etc.). The evolutionary relationship of CARM1 protein among different species is also shown in the phylogenetic tree data (Fig. S1b).

**Fig. 2:**
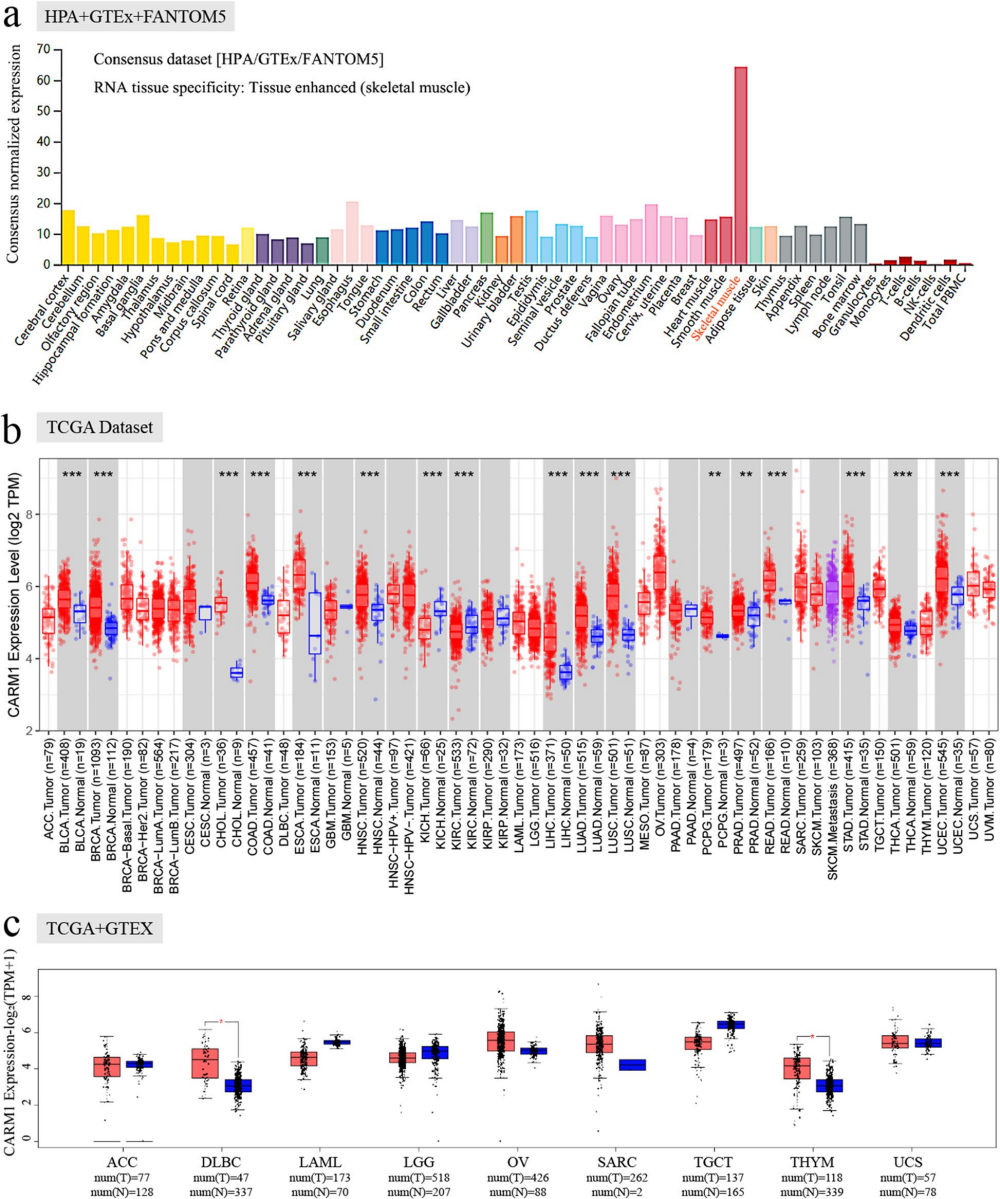
Expression profile of CARM1. **a** CARM1 expression in normal tissues. **b** The expression of CARM1 in tumors and normal tissues from TCGA project were compared by TIMER2. *P < 0.05, **P < 0.01, ***P < 0.001. **c** For the types of ACC, DLBC, LGG, OV, SARC, TGCT, THYM and UCS in TCGA project, the corresponding normal tissues in GTEx database were used as controls. * P < 0.05

Firstly, the physiologic CARM1 gene expression levels across normal tissues were observed combining HPA, GTEx and Function annotation of the mammalian genome 5 (FANTOM5) datasets. As shown in Fig. 2a, CARM1 expression is the highest in skeletal muscle with high RNA tissue specificity, while other detected tissues express relatively low level of CARM1, especially the blood cell lineage. When analyzing the expression of CARM1 in different blood cells, a low RNA blood cell specificity could be observed (Fig. S2b, see Additional file 2). The CARM1 expression levels in various cancer cell lines were also analyzed. The result shows that all cancers expressed CARM1, with the highest expression level in ovarian cancer, followed by endometrial cancer and colorectal cancer (Fig. S2a).

Next, the TIMER2 approach was applied to compare the expression difference of CARM1 between various cancer types and corresponding normal tissues. Among them, primary cancers show significantly higher expression levels than normal tissues in bladder urothelial carcinoma (BLCA), breast invasive carcinoma (BRCA), cholangio carcinoma (CHOL), colon adenocarcinoma (COAD), esophageal carcinoma (ESCA), head and neck squamous cell carcinoma (HNSC), liver hepatocellular carcinoma (LIHC), lung adenocarcinoma (LUAD), lung squamous cell carcinoma (LUSC), rectum adenocarcinoma (READ), stomach adenocarcinoma (STAD), thyroid carcinoma (THCA), uterine corpus endometrial carcinoma (UCEC) (*P* < 0.001), and pheochromocytoma and paraganglioma (PCPG), prostate adenocarcinoma (PRAD) (*P* < 0.01). In contrast, CARM1 is downregulated in tumor relative to normal tissues in kidney Chromophobe (KICH) and kidney renal clear cell carcinoma (KIRC) (*P* < 0.001) (Fig. 2b). After combining the normal tissue of the GTEx dataset as controls, the diffuse large B-cell lymphoma (DLBC) and thymoma (THYM) cohorts show upregulated expression levels, while there is no significant difference between the remaining tumor types and the corresponding normal tissues (Fig. 2c).

**Fig. 3:**
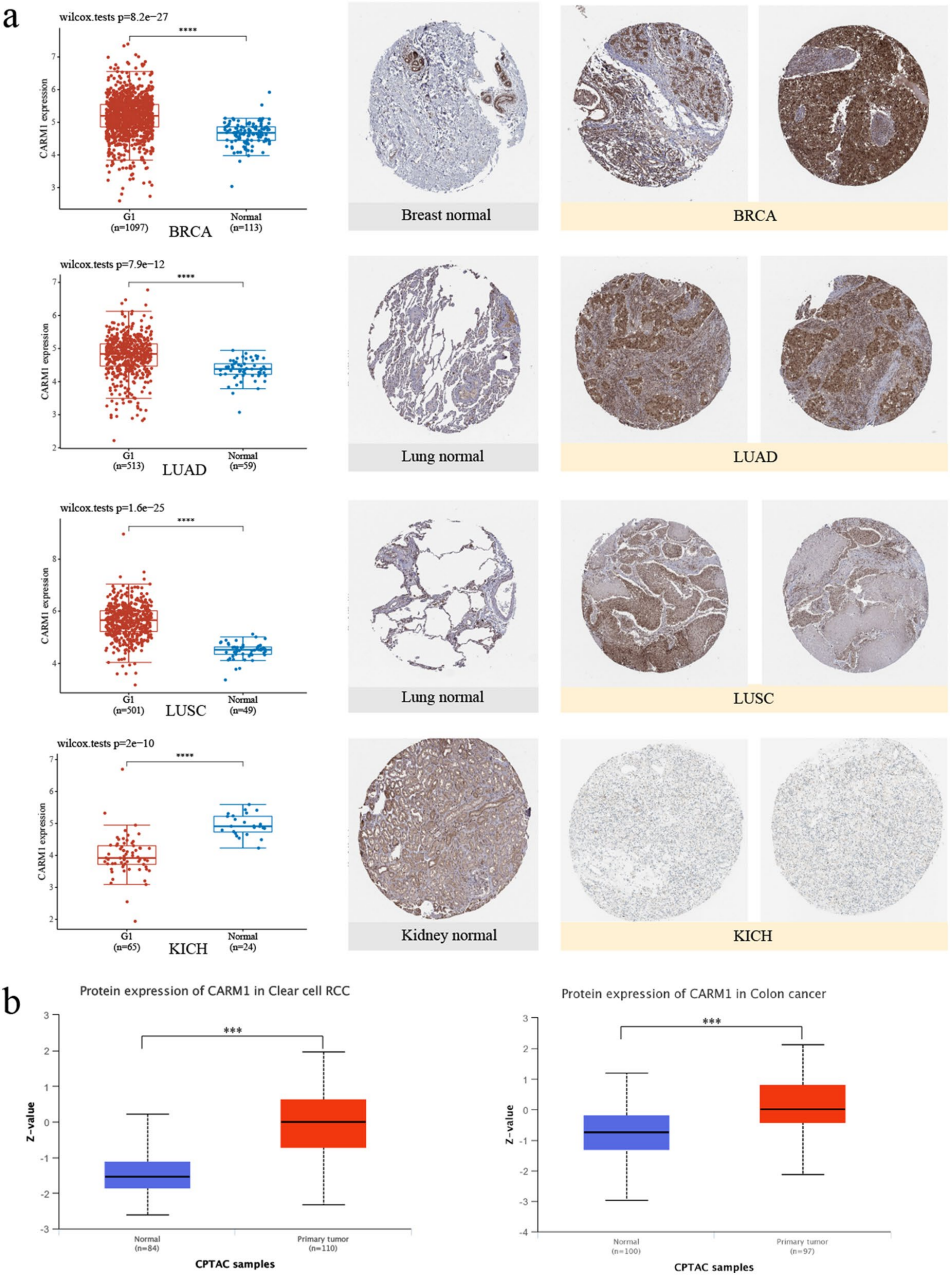
Analysis of CARM1 total protein expression data. **a** Comparison of CARM1 gene expression data from TCGA (left) with IHC results of HPA (right). The CARM1 RNA expression is up-regulated in BRCA, LUAD, LUSC and down-regulated in KICH, which is consistent with the results of IHC. **b** Data from CPTAC dataset indicate KIRC and colon cancer samples express higher level of CARM1 total protein than normal tissues. ***P < 0.001

In addition, HPA, TCGA and CPTAC datasets were used to evaluate CARM1 expression at protein level. We obtained the immunohistochemistry (IHC) results from HPA and compared them with the CARM1 gene expression data provided by TCGA. As shown in Fig. 3a, the analysis results from the two databases are basically consistent. The staining results of BRCA, LUAD, LUSC present strong or medium CARM1 staining, while the corresponding normal tissues show low or moderate staining. On the contrary, normal kidney tissues have low or moderate staining, while KICH samples have no CARM1 staining. Furthermore, the results of the CPTAC dataset indicate higher expression of CARM1 protein in the primary tissues of KIRC and colon cancer than in normal tissues (Fig. 3b), and increase from grade I to grade II in KIRC patients (Fig. S2c). It is noteworthy that although there are no significant correlations of protein expression between primary tissues of breast cancer, ovarian cancer, lung adenocarcinoma, UCEC and related normal tissues, the expression of protein in normal and other subtypes of breast cancer is significantly higher than that of luminal subtype. In addition, the CARM1 protein expression level of 21–40 years old group in ovarian cancer patients is up-regulated compared with other age groups, which may be a potential feature of this group.

**Fig. 4:**
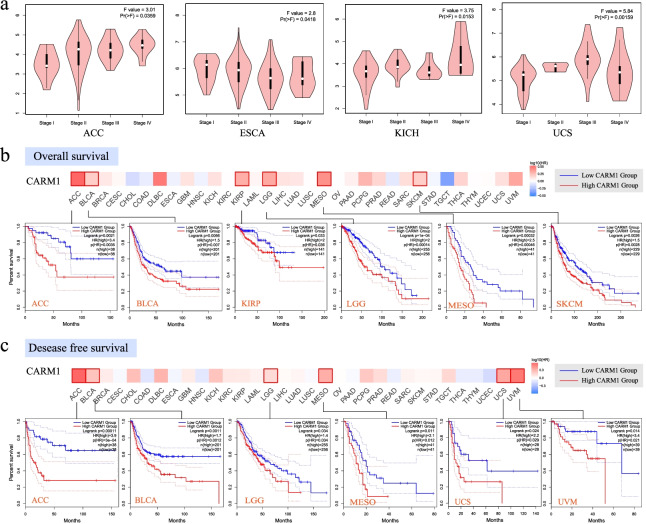
Correlation analysis of CARM1 gene expression with different pathological stages and survival prognosis across cancers in TCGA. **a** Based on data from TCGA, CARM1 gene expression levels were analyzed according to the main pathological stages. Log_2_ (TPM + 1) was applied for log-scale. GEPIA2 tool was used to perform overall survival (**b**) and disease-free survival (**c**) analysis of cancers in TCGA according to CARM1 gene expression. The survival map and Kaplan-Meier curve of positive results are given

Using the “Pathological Stage Plot” module of GEPIA2, we also found that the expression of CARM1 is related to the pathological stages of the following carcinomas, comprising ACC (adrenocortical carcinoma), ESCA, KICH and UCS (uterine carcinosarcoma) (Fig. 4a, all *P < 0.05*), but no significant difference is observed in other tumors (Fig. S3, see Additional file 3).

### Prognostic value of CARM1 in pan-cancers

To explore the correlation between CARM1 expression and prognosis of patients with different tumors, TCGA and GEO were used and cancer cases were divided into high-expression and low-expression groups according to the expression levels of CARM1. As shown in Fig. 4b, highly expressed CARM1 is linked to poor prognosis of overall survival (OS) for cancers such as ACC (*P = 0.0021*), BLCA (*P = 0.0066*), KIRP (kidney renal papillary cell carcinoma, *P = 0.033*), LGG (Brain lower Grade Glioma, *P = 0.00014*), MESO (Mesothelioma, *P = 0.00046*), SKCM (skin cutaneous melanoma, *P = 0.0026*). Analysis results of DFS presented a correlation between high CARM1 expression and poor prognosis of ACC (*P = 0.00011*), BLCA (*P = 0.0012*), LGG (*P = 0.034*), MESO (*P = 0.012*), UCS (*P = 0.029*) and UVM (uveal melanoma, *P = 0.021*) (Fig. 4c).

Furthermore, Kaplan-Meier Plotter tool was also used to identify the prognostic value of CARM1 in the five types of tumors shown in Fig. S4 (see Additional file 8), which manifest a correlation between high expression CARM1 and poor OS, PPS, FP prognosis for gastric cancer and lung cancer. As for ovarian cancer, low CARM1 expression is related to poor PFS, while the relationship between CARM1 expression and OS, PPS prognosis are not detected. Additionally, CARM1 is a high-risk gene in breast cancer (OS, *P = 0.019*; DMFS, *P = 0.00033*; PPS, *P = 0.0038*), while it is a low-risk gene in liver cancer (DSS, *P = 0.04*; RFS, *P = 0.033*; PFS, *P = 0.013*).

We also performed a subgroup survival analysis using selected clinical factors and observed different conclusions. Significantly, highly expressed CARM1 is linked to poor prognosis for estrogen receptor (ER) positive subgroup of breast cancer cases. As for patients in grade II or lymph node negative status, CARM1 overexpression may be a poor prognostic factor (Table 1). For gastric cancer patients with lymph node metastasis, highly expressed CARM1 is associated with poor OS, FP and PPS prognosis (Table S1, see Additional file 4). Notably, consistent with the overall analysis results of liver cancer cases aforementioned, CARM1 overexpression is a beneficial prognostic factor in most subgroup analyses, especially in patients with hepatitis virus infection or in low grade, and it may turn into a deleterious prognostic factor when the disease developed into high grade (Table S2, see Additional file 5). More details about prognosis of these five tumors can be found in Table 1, Table S1-S4 (see Additional files 4, 5, 6, 7).

**Table 1:** Subgroup analysis on the correlation of CARM1 expression and prognosis of breast cancer cases

Factor	Subgroup	Sample size	OS		RFS		DMFS		PPS	
			HR	*P*	HR	*P*	HR	*P*	HR	*P*
**ER status-IHC**	ER positive	3499	1.45	**0.028**	1.25	**0.0068**	1.26	0.26	1.84	**0.0023**
	ER negative	2168	1.28	0.17	1.2	0.095	1.53	**0.0021**	1.3	0.3
**ER status-array**	ER positive	5526	1.32	0.062	1.15	**0.042**	1.33	**0.0036**	1.52	**0.013**
	ER negative	2009	1.27	0.13	0.77	***0.013***	1.22	0.14	1.83	**0.012**
**TP53 status**	mutated	272	0.71	0.33	0.64	0.066	0.38	***0.02***	5.55	**0.0015**
	Wild type	388	0.65	0.17	1.68	**0.02**	0.47	***0.035***	2.08	**0.048**
**PR status**	PR positive	1559	1.52	0.27	1.61	**0.0025**	1.46	0.11	4.98	0.085
	PR negative	1989	0.54	***0.042***	1.14	0.25	1.37	**0.038**	0.32	***0.022***
**HER2 status**	HER2 positive	1273	1.29	0.18	0.79	***0.047***	1.77	***0.00077***	1.27	0.31
	HER2 negative	6262	1.28	**0.03**	1.11	0.092	1.26	**0.01**	1.48	**0.0039**
**Grade**	Grade 1	576	0.24	***0.041***	1.59	0.18	4.89	**0.018**	0.5	0.23
	Grade 2	1795	1.63	**0.021**	1.41	**0.0037**	1.38	**0.031**	1.97	**0.0055**
	Grade 3	2058	1.34	0.073	1.18	0.085	1.26	0.084	1.75	**0.007**
**Intrinsic subtype**	Basal	1494	1.49	**0.038**	0.79	0.068	1.25	0.16	2.01	**0.022**
	Luminal A	3511	1.32	0.16	0.88	0.12	1.31	**0.038**	1.77	**0.014**
	Luminal B	2015	1.36	0.083	1.16	0.11	1.5	**0.0071**	1.56	0.12
	HER2+	515	0.71	0.25	0.62	***0.0079***	1.54	0.086	1.81	0.12
**Lymph node status**	Lymph node positive	2153	1.2	0.3	0.86	0.12	0.83	0.19	1.77	**0.0055**
	Lymph node negative	2829	1.71	**0.018**	1.4	**0.00011**	1.51	**0.001**	1.66	**0.017**
**Pietenpol subtype**	Basal-like 1	418	2.02	0.1	0.53	***0.014***	0.7	0.26	2.39	0.2
	Basal-like 2	165	4.51	**0.0012**	0.68	0.22	1.49	0.3		
	immunomodulatory	462	0.66	0.3	0.71	0.21	2.02	**0.05**	0.27	***0.035***
	Mesenchymal	382	0.57	0.17	0.58	***0.03***	1.72	0.073	3.75	**0.033**
	Mesenchymal stem-like	201	0.32	***0.024***	0.54	0.11	1.76	0.28		
	Luminal androgen receptor	413	0.8	0.46	0.71	0.094	1.89	**0.043**	1.67	0.26

*ER* Estrogen receptor, *PR* Progesterone receptor, *HER2* human epidermal growth factor receptor 2, *NA* not available data, *HR* hazard ratio

The *P value* marked bold indicates that the prognosis of the low expression group is better than that of the high expression group, while the *P value* marked bold and italic indicates that the prognosis of the high expression group is better than that of the low expression group. NS, P > 0.05; * P < 0.05; ** P < 0.01; *** P < 0.001

### Genetic alteration analysis of CARM1 across cancers

**Fig. 5:**
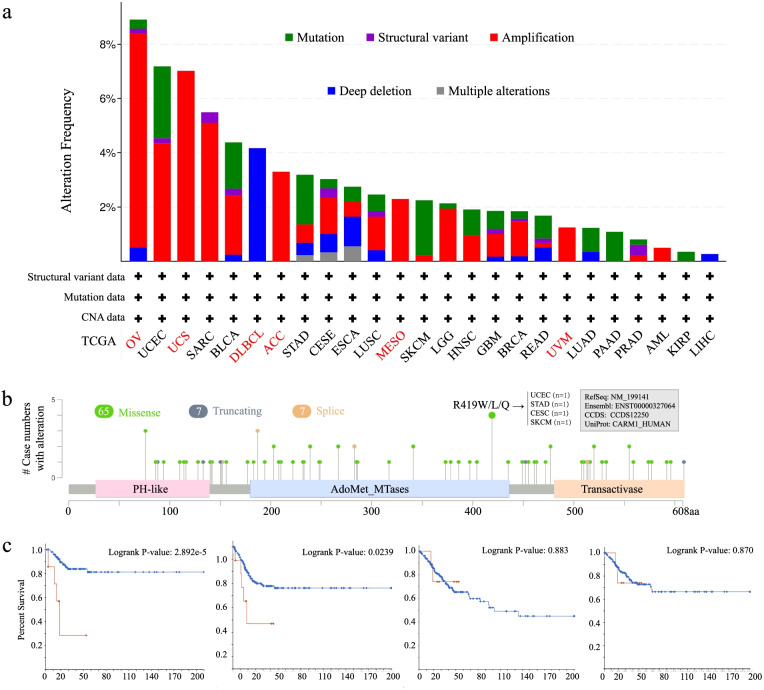
Mutation features of CARM1 in different cancers of TCGA. The cBioPortal tool was used to analyze the mutation features of CARM1. **a** The alteration frequency of different mutation types of CARM1 gene in different cancers. **b** The mutation types, sites and case number of the CARM1 genetic alteration were further presented. **c** Potential correlation between mutation status and DFS, PFS, OS and DSS rate of CESC. DFS, disease-free survival; PFS, progress-free survival; OS, overall survival; DSS, disease-specific survival

Gene alteration features of different cancers in TCGA were further investigated using the cBioPortal tool. Among all cancers, ovarian cancers present the highest alteration frequency of CARM1 (> 8%) with “amplification” as the primary type (Fig. 5a). It is worth noting that all cases of uterine carcinosarcoma (~ 7%), adrenocortical carcinoma (~ 3.5%) and mesothelioma (> 2%) with gene variation have “amplification” mutation type, while all cases of DLBCL (> 4%) have copy number deletion of CARM1. Figure 5b further shows the types, loci and number of cases of CARM1 gene variations. As the main type of gene change, there are 65 missenses in CARM1, among which 419 sites in the methyltransferase domain had the maximum R (arginine) mutations, translation from R to W (tryptophan), L (leucine) and Q (glutamine), respectively. Moreover, the potential association between CARM1 genetic alterations and clinical prognosis was analyzed in various cancers cases (Table 2). Compared with the cases with CARM1 change, Cervical squamous cell carcinoma and endocervical adenocarcinoma (CESC) cases without altered CARM1 show better prognosis in PFS (*P = 0.0239*) and DFS (*P = 2.892e-5*), but not OS (*P = 0.883*) and DSS (*P = 0.870*). These results have been shown in Fig. 5c. In addition, the prognosis of the group without genetic changes is significantly better than that of the relative group in COAD.

**Table 2:** Summary of CARM1 genetic alteration and clinical survival prognosis in various cancers cases

Tumor types	Sample size	*P*			
		OS	PFS	DSS	DFS
BLCA	562	0.743	NA	NA	**0.0465**
BRCA	963	0.793	NA	NA	0.469
CESC	278	0.883	**0.0239**	0.87	**0.00003**
COAD	436	0.521	**0.0202**	0.845	**0.0314**
SKCM	363	0.077	0.191	0.145	NA
GBM	378	0.356	0.501	0.273	NA
HNSC	385	**0.0012**	NA	NA	NA
LUAD	230	0.334	NA	NA	0.055
LUSC	469	0.62	0.44	0.263	0.583
PRAD	489	0.515	0.29	0.667	**0.0432**
STAD	434	**0.0315**	0.277	0.132	0.448
UCS	509	0.535	0.147	0.298	0.263

*P value* derived from Log Rank test. The bold font indicates that the prognosis of the mutated group is worse than that of the non-mutated group. NS, P > 0.05; * P < 0.05; ** P < 0.01; *** P < 0.001

**Fig. 6:**
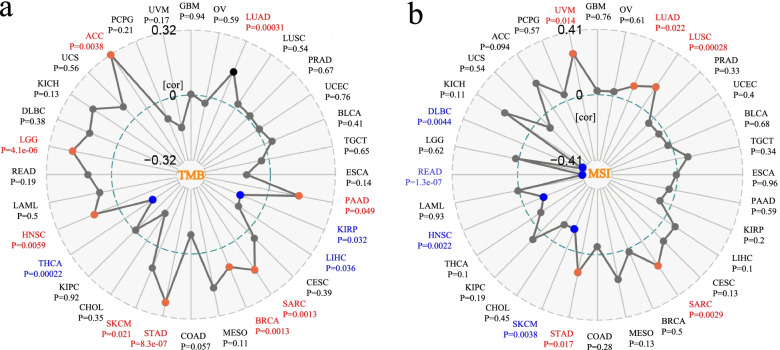
Correlation between CARM1 mRNA expression and TMB, MSI. Based on TCGA dataset, the potential correlation between CARM1 expression and TMB (**a**), MSI (**b**) is explored. TMB was calculated according to total mutation rate per million base pairs in each cancer, and MSI was counted by total incidence of deletion or insertion in repeating sequences per million base pairs. *The* partial correlation values are marked. Spearman correlation test, *P < 0.05* is considered significant. Red font represents positive correlation and blue font represents negative correlation

Serving as emerging prognostic and immunotherapeutic response biomarkers for a variety of tumors, the quantification of TMB and MSI have entered the exploratory stage of clinical transformation [26, 27]. Herein, we analyzed the correlation between CARM1 mRNA expression and TMB, MSI. As shown in Fig. 6a, there is a positive correlation between CARM1 expression and TMB for LUAD (*P = 0.00031*), pancreatic adenocarcinoma (PAAD, *P = 0.049*), SARC (*P = 0.0067*), BRCA (*P = 0.0013*), STAD (*P = 8.3e-07*), SKCM (*P = 0.021*), HNSC (*P = 0.0059*), LGG (*P = 4.1e-06*) and ACC (*P = 0.0038*), but a negative correlation for KIRP(*P = 0.032*), LIHC (*P = 0.036*), THCA (*P* = *0.00022*). As for MSI, CARM1 expression is also positively correlated with LUAD (*P = 0.022*), LUSC (*P = 0.00028*), SARC (sarcoma, *P = 0.0029*), STAD (*P = 0.017*) and UVM (*P = 0.014*), but is negatively correlated with that of SKCM (P = 0.0038), HNSC (*P = 0.0022*), READ (*P = 1.3e-07*) and DLBC (*P = 0.0044*). It should be noted that both TMB and MSI of LUAD, SARC and STAD are positively correlated with CARM1 expression, which deserves further study.

### CARM1 expression is related to DNA repair genes and methyltransferase expression in various tumor samples

**Fig. 7:**
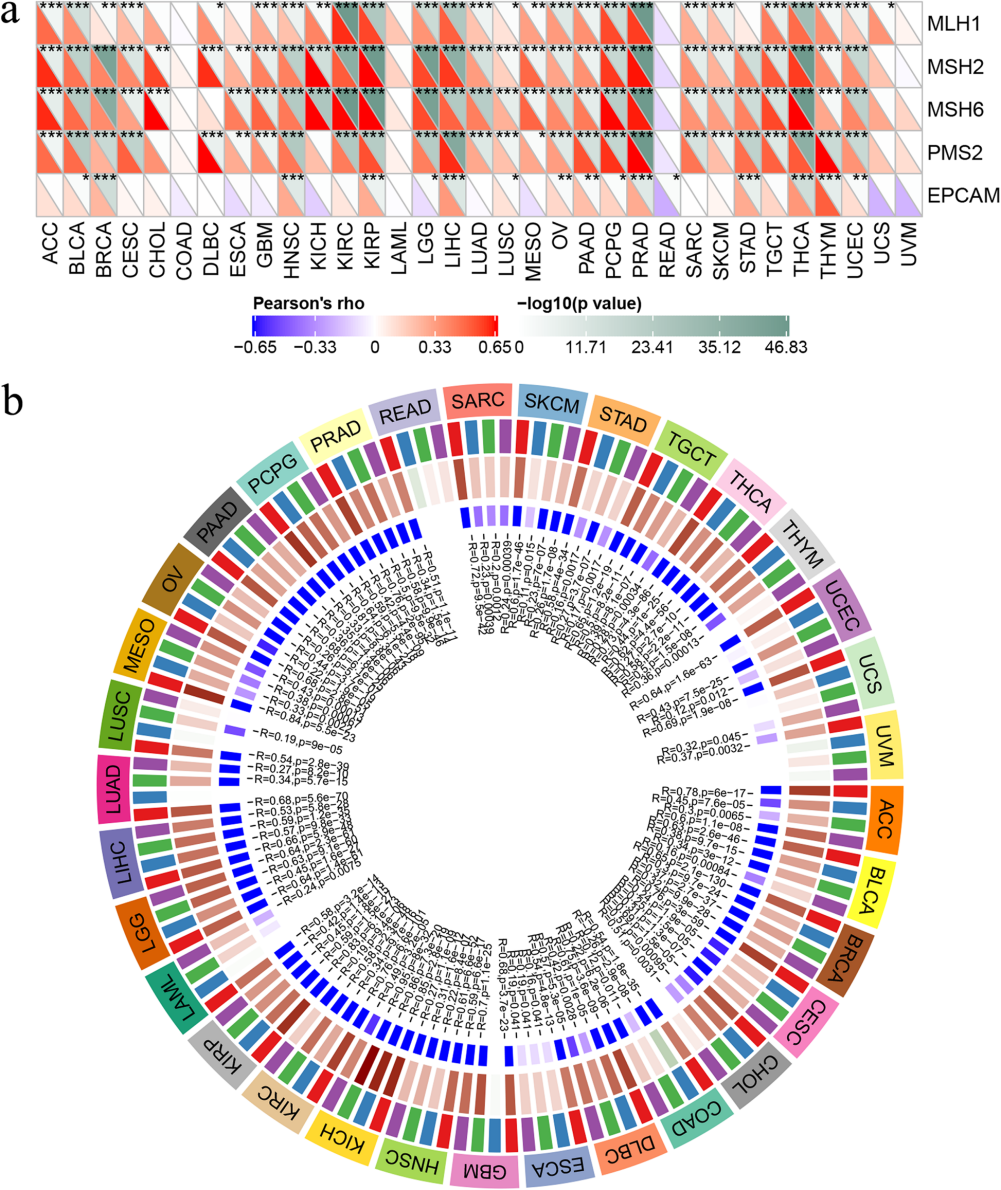
Correlation analysis of CARM1 expression with MMR genes mutation and methyltransferases genes expression. **a** Correlation between CARM1 mRNA expression and five major MMR genes mutation. The lower triangle in each block represents the coefficient calculated by Pearson’s correlation test, and the upper triangle represents log_10_ transformed *P*-value. * P < 0.05, ** P < 0.01, *** P < 0.001. **b** Co-expression analysis of CARM1 and methyltransferases. The outer circle represents different tumor types, the second circle represents the four methyltransferases (DNMT1: red, DNMT2: blue, Dnmt3a: green, DNMT3b: Purple), the third circle represents co-expression correlation coefficient, and the fourth circle represents *P value*. *P < 0.05* is considered significant

Correlation between mutation indexes TMB, MSI and CARM expression prompted us to further explore the potential relationship between CARM1 expression and tumorigenesis mechanism. MMRs, the intracellular mismatch repair mechanisms, the loss of function of its key genes will lead to unrepairable DNA replication errors, and then result in somatic mutations [28]. Here, utilizing TCGA expression profile data, we evaluated the relationship between CARM1 expression and mutation of five major MMRs genes including MLH1, MSH2, MSH6, PMS2, EPCAM. Except COAD, AML (acute myeloid leukemia), READ, UCS and UVM, the expression of CARM1 is positively correlated with MMRs genes mutation in almost all types of tumors from TCGA, and the results of MLH1, MSH2, MSH6 and PMS2 are more significant (Fig. 7a).

As a form of DNA chemical modification involving the transfer of methyl group onto the C5 position of the cytosine to form 5-methylcytosine under the action of DNA methyltransferase, DNA methylation can change genetic performance without changing DNA sequence, which can change chromatin structure, DNA conformation, DNA stability and the interaction between DNA and protein, so as to regulate gene expression [29]. Here, analysis of the correlation between CARM1 and four methyltransferases (DNMT1, DNMT2, DNMT3a, DNMT3b) expression was conducted for each tumor to explore whether CARM1 expression is related to epigenetics. The result shows that CARM1 and methyltransferases are significantly co-expressed in almost all tumors (Fig. 7b), which is an interesting phenomenon worth further exploring.

### Correlation between TME and CARM1 expression

#### Correlation between tumor-infiltrating immune cells and CARM1 expression

Tumor microenvironment (TME) is an environment conducive to tumor cell survival established by tumor cells that evade early immune surveillance by remodeling local immune cells and stromal cells, and a full understanding of TME provides us with valuable clues to develop more effective therapeutic strategies. Currently, there is still a lack of research on the association of CARM1 methylation and immune cell infiltration. In an impressive study, CARM1 inactivation was found to activate innate immunity in melanoma resistant cell lines with high CARM1 expression, making them more sensitive to T cell immunity and immune checkpoint blockade [30]. Moreover, it is eye-catching that CARM1-KO T cells exert more effective antitumor effects than wild-type, indicating that CARM1 inhibition enables immunotherapy of resistant tumors by dual effects on tumor cells and T cells, which has great clinical translational value. And the analysis results presented below in this study also manifest there is a large correlation between immune cells infiltration and CARM1 expression in the TME of some tumors, pointing out that investigating the role of CARM1 in the field of tumor immunity in-depth is a direction worth exploring.

**Fig. 8:**
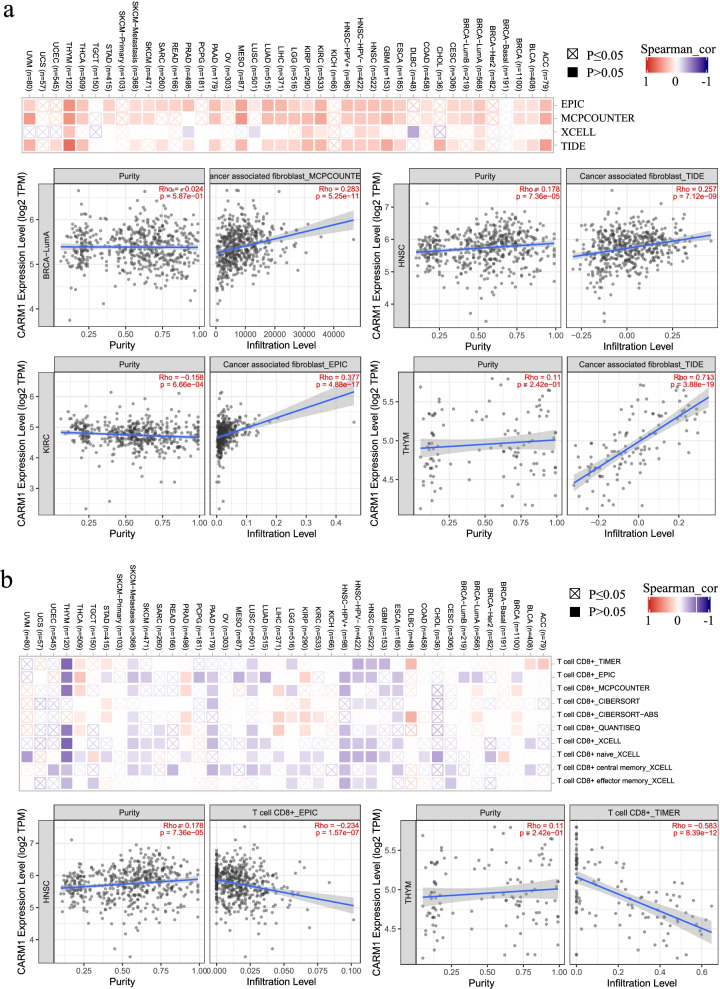
Different algorithms were used to explore the potential correlation between CARM1 expression and immune infiltration across all types of cancer in TCGA. **a** Correlation heat map and representative scatterplots of cancer-associated fibroblasts infiltration, indicating that CARM1 expression is positively correlated with the cancer-related fibroblasts infiltration in most tumors, especially in ACC, KIRC, MESO, THYM and UVM. **b** Correlation heat map and representative scatterplots of CD8+ T cell, showing that CD8+ T cells infiltration is negatively correlated with CARM1 expression in ESCA, HNSC, LUSC, PAAD, SKCM and THYM. *P < 0.05* is considered significant

A growing number of researches show that tumor-infiltrating immune cells serve as a vital part in TME, affecting the occurrence and development of tumors significantly [31, 32]. It is of great significance to further explore the pan cancerous relationship between these immune cells and CARM1 expression. As a significant part of TME, cancer-associated fibroblasts in tumor stroma have been reported to be involved in regulating the function of a variety of tumor infiltrating immune cells [33]. Therefore, several algorithms were applied to investigate the relationship between CARM1 expression and cancer-associated fibroblasts infiltration. As shown in Fig. 8a, in most TCGA tumors, the expression of CARM1 is positively correlated with the infiltration of cancer-related fibroblasts, especially in ACC, KIRC, MESO, THYM and UVM. The representative scatterplots produced using one algorithm are also presented in the Figure.

Because tumor infiltrating lymphocytes are independent predictors of sentinel lymph node status and survival in cancer [32], analogous analysis on potential correlation between CD8^+^ T cells infiltration and CARM1 expression is also conducted. The results show that CD8^+^ T cells infiltration is negatively correlated with CARM1 expression in ESCA, HNSC, LUSC, PAAD, SKCM and THYM cases (Fig. 8b). Similar trend could be found between CARM1 expression and other immune cells infiltration in ESCA, LUSC, SKCM and THYM in Table 3, which presents more comprehensive details. On the contrary, a positive correlation trend could be observed in BLCA, BRCA, KIRC, KIRP, LGG, LIHC, LUAD, PAAD, PCPG, PRAD and THCA.

**Table 3:** Correlation analysis between CARM1 expression and other immune cells infiltration

Tumor type	B cell	CD4+ T cell	CD8+ T cell	Neutrophil	Macrophage	Dendritic
ACC	0.357/***	−0.006/ns	−0.023/ns	0.149/0.19	0.047/ns	0.243/*
BLCA	−0.011/ns	0.017/ns	0.262/***	0.152/***	0.086/ns	0.209/***
BRCA	0.104/***	0.19/***	0.096/**	0.206/***	0.09/**	0.168/***
CESE	−0.065/ns	0.069/ns	−0.134/*	− 0.083/ns	− 0.178/**	− 0.022/ns
CHOL	0.139/ns	0.106/ns	−0.15/ns	0.459/**	0.267/ns	0.101/ns
COAD	− 0.157/***	0.141/**	− 0.267/***	− 0.012/ns	− 0.044/ns	− 0.003/ns
DLBC	0.089/ns	0.096/ns	− 0.504/**	0.4/*	0.192/ns	−0.035/ns
ESCA	−0.102/ns	−0.103/ns	− 0.295/***	−0.22/**	− 0.174/*	−0.128/ns
GBM	−0.014/ns	−0.11/ns	− 0.047/ns	−0.043/ns	− 0.036/ns	−0.091/ns
HNSC	−0.047/ns	−0.198/***	− 0.073/ns	0.046/ns	0.076/ns	0.065/ns
KICH	0.082/ns	0.065/ns	0.311/*	−0.091/ns	0.446/***	0.109/ns
KIRC	0.264/***	0.45/***	0.203/***	0.402/***	0..399/***	0.43/***
KIRP	0.117/*	0.097/ns	0.079/ns	0.187/**	0.01/ns	0.226/***
LGG	0.331/***	0.198/***	0.228/***	0.383/***	0.226/***	0.315/***
LIHC	0.309/***	0.441/ns	0.111/*	0.321/***	0.348/***	0.301/***
LUAD	−0.049/ns	0.235/***	−0.044/ns	0.179/***	0.113/*	0.151/***
LUSC	−0.147/***	−0.024/ns	− 0.302/***	−0.292/***	− 0.198/***	−0.249/***
MESO	0.266/*	0.073/ns	0.108/ns	−0.267/*	0.119/ns	0.306/**
OV	0.035/ns	−0.041/ns	−0.04/ns	− 0.049/ns	0.094/ns	0.006/ns
PAAD	0.204/**	0.094/ns	0.331/***	0.38/***	0.426/***	0.375/***
PCPG	0.106/ns	0.15/*	0.124/ns	0.227/**	0.313/***	0.179/*
PRAD	0.405/***	0.111/*	0.112/***	0.313/***	0.352/***	00.33/***
READ	−0.064/ns	0.125/ns	−0.346/***	−0.245/**	− 0.162/*	0.01/ns
SARC	−0.134/*	−0.333/***	− 0.139/*	−0.096/ns	− 0.217/***	−0.307/***
SKCM	−0.149/**	−0.004/*	− 0.208/***	−0.125/***	− 0.036/ns	0.144/**
STAD	−0.103/*	0.002/ns	−0.116/*	−0.1/ns	− 0.123/*	−0.072/ns
TGCT	0.119/ns	−0.28/***	0.137/ns	−0.252/**	0.024/ns	−0.009/ns
THCA	0.577/***	0.66/***	−0.343/***	0.458/***	0.504/***	0.403/***
THYM	−0.008/ns	−0.437/***	− 0.179/ns	0.252/**	0.005/ns	−0.255/**
UCEC	−0.097/*	−0.076/ns	− 0.075/ns	0.033/ns	− 0.148/***	−0.023/ns
UCS	−0.087/ns	−0.186/ns	0.048/ns	−0.002/ns	0.261/ns	0.335/*
UVM	−0.093/ns	0.034/ns	0.12/ns	−0.061/ns	0.032/ns	0.254/*

NS P > 0.05, * P < 0.05, ** P < 0.01, *** P < 0.001

#### Correlation between TME scores and CARM1 expression

**Fig. 9:**
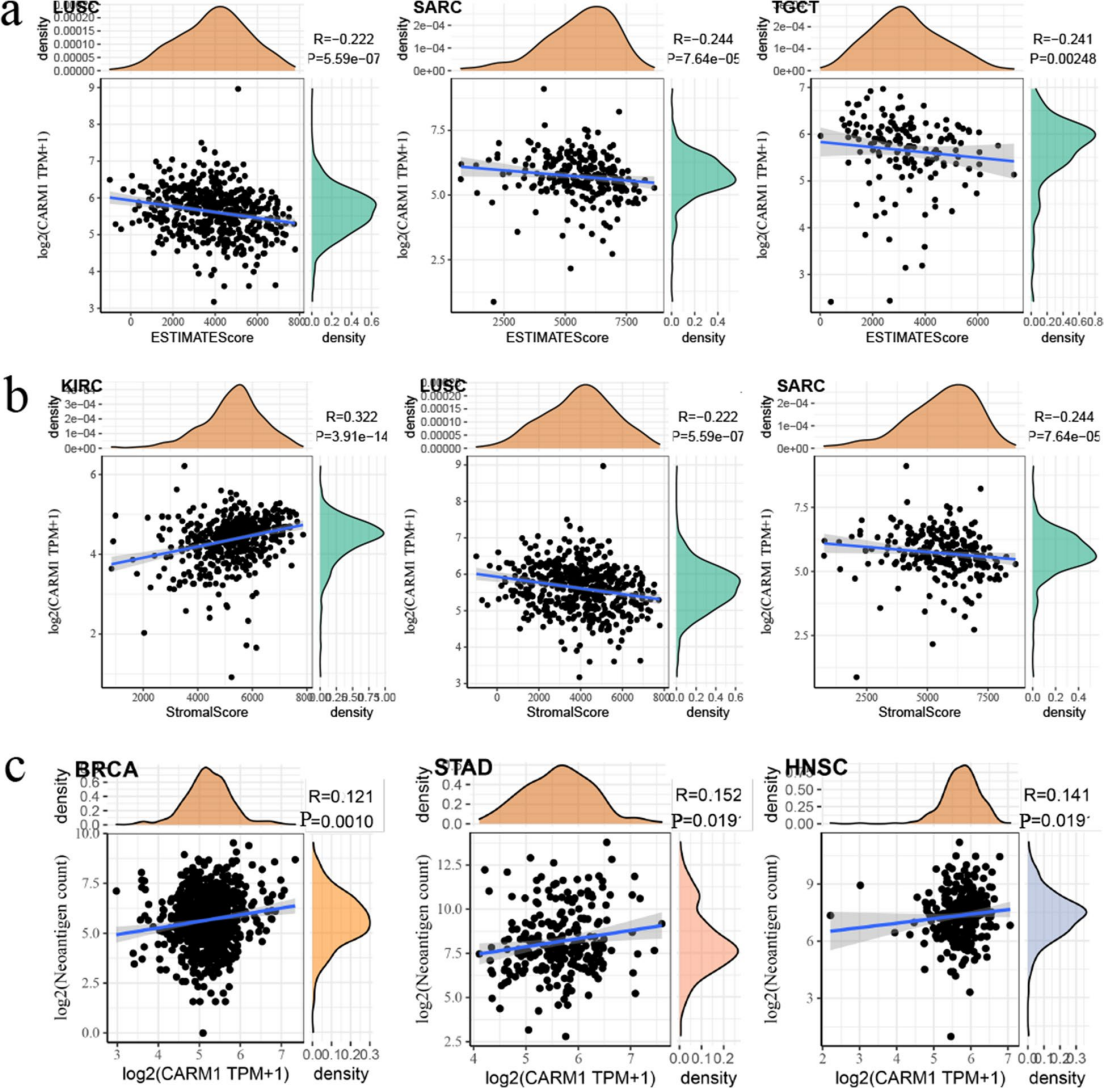
The top three tumors with the highest correlation coefficient between CARM1 expression and TME scores. **a** Negative correlation could be observed between CARM1 expression and immune estimate scores in LUSC, SARC, TGCT. **b** Correlation between CARM1 expression and stromal scores in KIRC, LUSC, SARC. **c** Positive correlation between CARM1 expression and the number of tumor immune neoantigens in BRCA, STAD, HNSC. Correlation coefficient R greater than zero indicates positive correlation, and less than zero indicates negative correlation. *P < 0.05* is considered significant

In order to conduct more in-depth research on the pan-cancer relationship between TME and CARM1 expression, ESTIMATE algorithm was used to analyze the relationship between stromal and immune scores and gene expression level among 33 tumors from TCGA. The results of the top three tumors with the highest correlation coefficient have been shown in Fig. 9, which reveal that CARM1 expression is significantly negatively correlated with immune scores in LUSC, SARC, testicular germ cell tumors (TGCT), indicating that the content of immune cells decreases while the level of CARM1 expression escalates (Fig. 9a). Similar results were also observed in the stromal score of LUSC and SARC, while the opposite correlation was shown in KIRC (Fig. 9b).

Genetic instability of tumor cells often leads to a large number of mutations, and the expression of nonsynonymous mutations could produce tumor specific antigens called tumor neoantigens [34]. Because they are not expressed in normal tissues, neoantigens have high immunogenicity and can activate T cells to trigger immune response, which have become a potential new target of tumor immunotherapy. Here, we counted the number of neoantigens in each tumor sample, and analyzed the relationship between them and the expression of CARM1. As shown in Fig. 9c, there is a significant positive correlation between CARM1 expression and the number of immune neoantigens in BRCA, STAD and HNSC, suggesting a new idea of immunotherapy.

#### Correlation between immune checkpoints and CARM1 expression

Tumor cells induce immunosuppression through various ways to achieve immune escape, and tumor immunotherapy is a treatment method to control and eliminate tumors by restarting and maintaining tumor immune cycle and restoring normal anti-tumor immune response, in which immune checkpoint inhibitor is an important aspect [35]. Herein, we also conducted a correlation analysis between CARM1 and checkpoint genes expression and found that CARM1 expression is highly correlated with CD276 in various cancer types (Fig. S5, see Additional file 9). Additionally, CARM1 expression has a certain correlation with the expression of multiple immune checkpoints in KICH, KIRC, KIRP, LGG, LIHC and THCA. In contrast, the expression of CARM1 is negatively correlated with most immune checkpoint molecules in SKCM and TGCT.

### CARM1-associated genes enrichment analysis

**Fig. 10:**
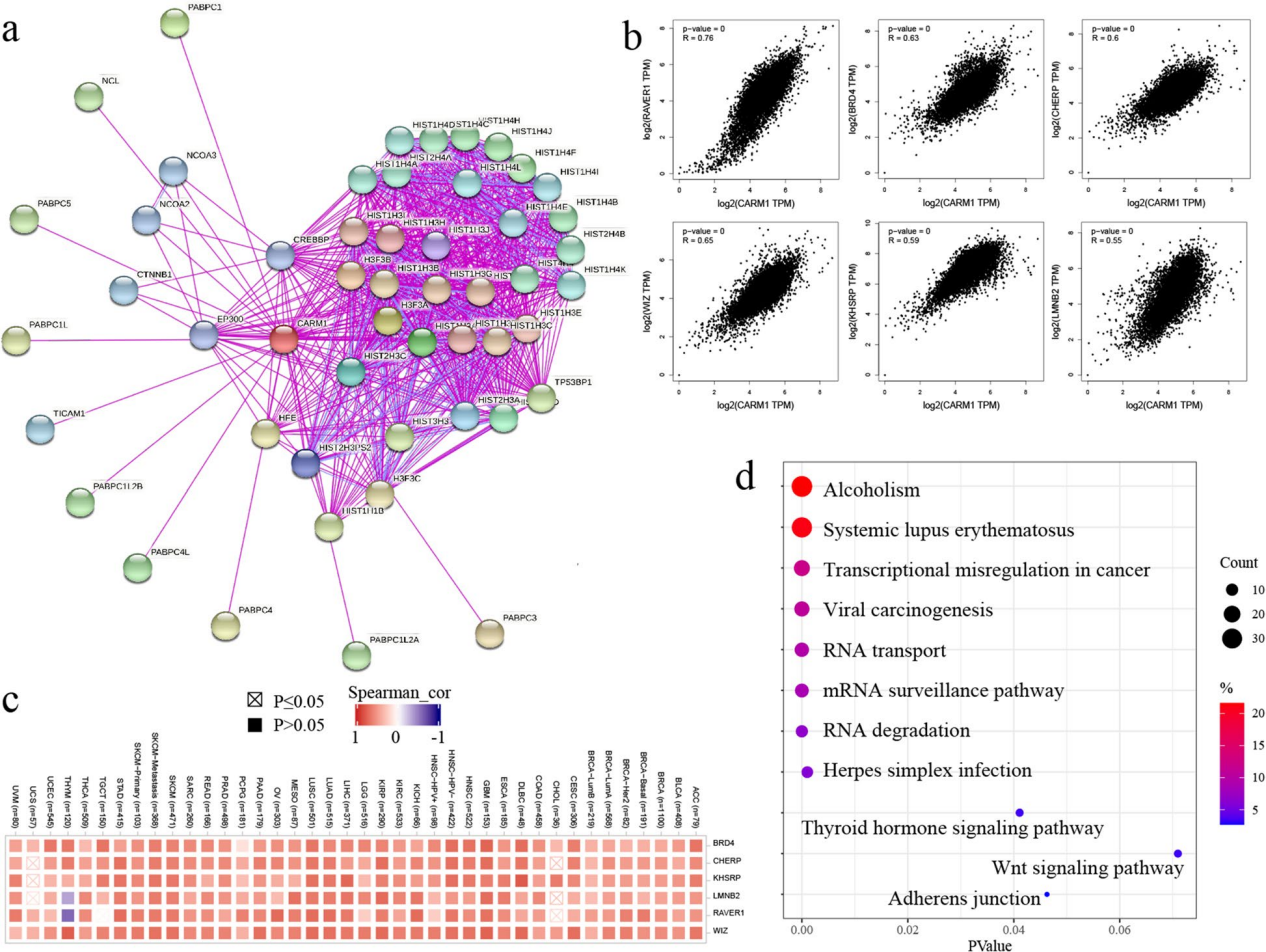
CARM1-associated genes enrichment analysis. **a** Based on STRING tool, the top 50 CARM1-binding proteins and the interaction network were obtained. **b** Using GEPIA2 method, the top 100 CARM1 related genes in TCGA project were gained, and the expression correlation between CARM1 and the top 6 target genes was analyzed. **c** Corresponding heatmap data was obtained utilizing TIMER2.0 online tool and further identifies CARM1 is positively correlated with the above 6 genes in most cancer types. **d** Combined with the binding and related proteins, KEGG enrichment analysis was conducted, showing clearly that the role of CARM1 in tumor pathogenesis may be related to transcriptional misregulation and viral carcinogenesis

In order to further study the biological significance of CARM1 gene in tumorigenesis, we screened CARM1 binding protein and expression related genes, and carried out a series of pathway enrichment analysis. STRING tool was applied to obtain the top 50 CARM1 binding proteins, which have been shown in the form of interaction network in Fig. 10a. Then, we used the GEPIA2 to acquire the first 100 genes related to CARM1 expression, and the top 6 genes with the highest correlation are shown in the form of scatter diagram in Fig. 10b. Corresponding heatmap data also indicate that CARM1 is positively correlated with the above 6 genes in most cancer types (Fig. 10c). Combined with the above two databases, we conducted KEGG enrichment analysis. The results show that the effect of CARM1 on tumor pathogenesis may be related to transcriptional misregulation and viral carcinogenesis (Fig. 10d).

## Discussion

CARM1 has been well known as a transcriptional coactivator, it has also been found to be crucial in regulation of metabolism, autophagy, RNA regulation and early mammalian development. In addition, increasing evidences indicate that CARM1 exerts an impact on the occurrence and development of tumors. After literature search, we found there is still a lack of research reports on pan-cancer analysis of CARM1. Therefore, based on the data from TCGA, CPTAC and other databases, we comprehensively detected the potential significance of CARM1 expression in various cancers from the perspectives of gene expression, gene alteration, immune microenvironment and related signaling pathways. Overexpression of CARM1 in most tumors was first verified, which is also associated with the pathological stage of some tumors, such as ACC, ESCA, KICH and UCS. Survival analysis results from GEPIA2 indicate that high expression of CARM1 is a significant adverse prognostic factor in ACC, BLCA, LGG, MESO, SKCM and other tumors. Overall, according to the analysis conducted by Kaplan Meier plotter, among the five tumors provided by the website, the low expression CARM1 group has a better clinical prognosis. However, subgroup analysis shows that the prognosis value of the CARM1 expression level vary in some subgroups. For example, the effect of CARM1 expression on prognosis in breast cancer is related to the state of HER2. In the HER2^−^ group, the prognosis of CARM1 low expression group is better than that of high expression group, while the prognosis of HER2^+^ subgroup is the opposite, which has been confirmed in previous studies [36]. In patients with hepatocellular carcinoma, high expression of CARM1 has a better prognosis in patients with hepatitis virus infection, while low expression of CARM1 is associated with favorable clinical prognosis of OS, FP and PPS, especially in high stage, specific TNM grading and pathological classification. Additionally, the analysis data obtained from cBioPortal tool shows that the mutation of CARM1 gene is related to the poor prognosis of colon cancer, which has been proved to be highly expressed CARM1 protein by CPTAC analysis tool. Therefore, on the premise of fully considering subgroup factors, CARM1 expression level is expected to be a good prognostic index. This study also analyzed a series of immune related factors of CARM1. Based on the results of immune cells infiltration, immune and matrix score as well as co-expression analysis of MMRs, methyltransferases genes and immune checkpoint genes, we found that CARM1 potentially affects the tumor immune microenvironment in most tumors, especially ACC, LUAD, LUSC, STAD, HNSC, THYM, etc. It is a direction worth exploring to clarify how CARM1 affects tumor immunity.

Our study conducts a comprehensive analysis of CARM1 in pan-cancer, which could provide clues for detecting its prognostic value and potential immunological function in tumor therapy. Information on various indicators suggesting the potential significance of CARM1 in different tumors has been summarized in Table 4, where the overall impact and conclusions about CARM1 on a certain tumor can be quickly found. Nevertheless, there are still some limitations in the present study. Although the correlation analysis between the gene expression of CARM1 and immune related factors implies the relevancy between them, it is not enough to capture detailed interaction. The concrete mechanisms of CARM1 affecting tumor immune microenvironment still needs further experimental verification.

**Table 4:** Summary of various indicators implying the potential significance of CARM1 in different tumors

Tumor type	CARM1 RNA expression	CARM1 protein expression	High vs Low CARM1 expression group		Predominant genomic alterations	Survival (altered vs unaltered group)	Correlation with CARM1 mRNA expression			Prognostic value assessment	Evidence from in vitro*/*in vivo cancer model studies	
			OS	DFS			TMB	MSI	Tumor neoantigen		Researches on CARM1 in this cancer	Effect of CARM1 on this cancer
ACC	NS	Related to the pathological stages	**	***	Amplification (3.3%)	NA	PC **	NS	NS	Unfavorable	NA	NA
BLCA	Up	Correlated with subtypes	**	**	Amplification (2.19%)	DFS*	NS	NS	NS	Unfavorable	NA	NA
BRCA	Up	Up	**^#^	NA	Amplification (1.29%)	NS	PC **	NS	PC **	Unfavorable, potentially associated with HER2 expression	[13], [16], [17], [18]	Pro-tumorgenic
CESE	NS	NA	NA	NA	Amplification (1.35%)	PFS*/DFS***	NS	NS	NS	Unfavorable	NA	NA
CHOL	Up	NA	NA	NA	NA	NS	NS	NS	NS	Not prognostic	NA	NA
COAD	Up	Up	NA	NA	Mutation (0.84%)	PFS*/DFS*	NS	NS	NS	Unfavorable	NA	NA
DLBC	Up	NA	NA	NA	Deep deletion (4.17%)	NA	NS	NC **	NA	Not prognostic	[19]	Pro-tumorgenic
ESCA	Up	Related to the pathological stages	NA	NA	Deep deletion (1.1%)	NA	NS	NS	NA	Not enough evidence	NA	NA
GBM	NS	NA	*	NA	Amplification (0.84%)	NS	NS	NS	NS	Not enough evidence	[20]	Pro-proliferative
HNSC	Up	NA	*	NA	Mutation/Amplification (0.96%)	OS**	PC **	NC **	NA	Unfavorable, related to TME	NA	NA
KICH	Down	Down	NA	NA	NA	NA	NS	NS	NA	Not enough evidence	NA	NA
KIRC	Down	Up	***	NA	Mutation (0.35%)	NA	NS	NS	NS	Unfavorable, related to TME	NA	NA
KIRP	NS	NA	***	NA	NA	NA	NC *	NS	NS	Unfavorable, related to TME	NA	NA
LAML	NS	NA	NA	NA	Amplification (0.5%)	NA	NS	NS	NA	Not prognostic	[21]	Pro-tumorgenic
LGG	NS	NA	***	**	Amplification (1.95%)	NA	PC ***	NS	NS	Unfavorable	NA	NA
LIHC	Up	NA	NS^#^	NA	Deep deletion (0.27%)	NA	NC *	NS	NS	Favorable in some subgroups	[6], [22]	Pro-proliferative; Antiproliferative
LUAD	Up	Up	***	NA	Mutation (0.88%)	NS	PC ***	PC *	NS	Unfavorable	[23]	Pro-proliferative
LUSC	Up	Up	***	NA	Amplification (1.23%)	NS	NS	PC ***	NS	Unfavorable, related to TME	NA	NA
MESO	NA	NA	***	*	Amplification (2.3%)	NA	NS	NS	NA	Unfavorable	NA	NA
OV	NS	NA	NS^#^	NA	Amplification (7.88%)	NA	NS	NS	NS	Vary from subgroups	NA	NA
PAAD	NS	NA	*	NA	Mutation (1.09%)	NA	PC *	NS	NA	Not enough evidence	[5]	Antiproliferative
PCPG	Up	NA	NA	NA	NA	NA	NS	NS	NA	Not prognostic	NA	NA
PRAD	Up	NA	NA	NA	Structural variant (0.4%)	DFS*	NS	NS	NS	Not enough evidence	[24]	Pro-tumorigenic
READ	Up	NA	NA	NA	NA	NA	NS	NC ***	NS	Not prognostic	NA	NA
SARC	NS	NA	NA	NA	Amplification (5.1%)	NA	PC **	PC **	NA	Not prognostic	[25]	Pro-proliferative
SKCM	NS	NA	**	NA	Mutation (2.03%)	NS	PC *	NC **	NS	Not enough evidence	NA	NA
STAD	Up	NA	NA	NA	Mutation (1.82%)	OS*	PC ***	PC *	PC *	Not enough evidence	NA	NA
TGCT	NS	NA	*	NA	NA	NA	NS	NS	NA	Not enough evidence	NA	NA
THCA	Up	NA	NS	NA	NA	NA	NC ***	NS	NS	Not prognostic, related to TME	NA	NA
THYM	Up	NA	NA	NA	NA	NA	NS	NS	NA	Related to TME	NA	NA
UCEC	Up	NS	NA	NA	Amplification (4.35%)	NA	NS	NS	NS	Not prognostic	NA	NA
UCS	NS	Related to the pathological stages	NA	*	Amplification (7.02%)	NS	NS	NS	NA	Not enough evidence	NA	NA
UVM	NA	NA	NA	*	Amplification (1.25%)	NA	NS	PC *	NA	Not enough evidence	NA	NA

*NS* not significance, *P > 0.05*, *NA* not available, *PC* positive correlation, *NC* negative correlation

Superscript # indicates that more detailed subgroup analysis data on this index are provided in the supplementary material. In most tumors, CARM1 expression is correlated with MMRs and methyltransferases gene expression, which can be seen in Fig. 7. Considering that there are too many content about the correlation between CARM1 expression and immune cell infiltration, detailed information can be found in Table 3 and will not be summarized here. NS, P > 0.05; * P < 0.05; ** P < 0.01; *** P < 0.001

## Conclusions

In the light of big data analysis based on multiple databases, we revealed that the expression level and mutation degree of CARM1 are significantly related with clinical prognosis of patients with various tumors, indicating that CARM1 is expected to become an effective prognostic index. In the process of exploring mechanisms of CARM1 involved in tumor progression, we correlated probable causes from the perspective of several immune related elements and signaling pathways, which would be conducive to provide clues to support further molecular mechanism exploration, and may furnish an immune based antitumor strategy to provide a reference for more accurate and personalized immunotherapy in the future.

## Methods

### Acquisition of gene information and protein structure

The detailed genomic location information of CARM1 gene was obtained by querying UCSC website (http://genome.ucsc.edu/). Then, the protein structure diagrams of CARM1 containing conserved regions in different species were gained and analyzed by using the Homologene module in NCBI website (https://www.ncbi.nlm.nih.gov/homologene/). Additionally, the phylogenetic tree of CARM1 in different species was also acquired by using the COBALT online tool of NCBI (https://www.ncbi.nlm.nih.gov/tools/cobalt/).

### Gene expression analysis at mRNA level

The expression data in different tissues, blood cells and tumor cell lines under physiological conditions were obtained by inputting “CARM1” in HPA database (https://www.proteinatlas.org/humanproteome/pathology). Using the “Gene_DE” module of TIMER2 (tumor immune estimation resource, version 2) website (http://timer.cistrome.org/), the expression difference of CARM1 between tumors and corresponding normal tissues in TCGA project was observed. As for tumors without normal control, “Expression analysis-Box Plots” module of GEPIA (Gene Expression Profiling Interactive Analysis, version 2) was applied to acquire expression data from GTEx (Genotype-Tissue expression) database, under the settings of “*P-value* cutoff = 0.01, log2FC (fold change) cutoff =1” and “Match TCGA normal and GTEx data” (http://gepia2.cancer-pku.cn/) [37]. We also utilize the “pathological staging map” module of GEPIA2 to obtain the violin plot of CARM1 expression in different pathological stages (stage I, II, III and IV) of all TCGA tumors.

### Gene expression analysis at protein level

To evaluate the CARM1 expression difference from the protein expression level, immunohistochemistry images of tumor tissues and corresponding normal tissues were downloaded from HPA and analyzed. Further, we obtained CPTAC (clinical proteome tumor analysis alliance) dataset from the UALCAN portal (http://ualcan.path.uab.edu/analysis-prot.html) and conducted protein expression analysis of various tumors.

### Survival prognosis analysis

Utilizing the “Survival Map” and “Survival Analysis” module of GEPIA 2, the OS (overall survival) and DFS (disease-free survival) significance map data and survival map of CARM1 in all TCGA tumors can be obtained respectively. Cutoff-high (50%) and cutoff-low (50%) values were used as the expression thresholds to split the high-expression and low-expression cohorts. The association between CARM1 expression and survival in pan-cancer was also verified by Kaplan-Meier Plotter (https://kmplot.com/analysis/), which pools the different GEO datasets for a series of analyses of OS, DMFS (distant metastasis-free survival), RFS (relapse-free survival), PPS (post-progression survival), FP (first progression), DSS (disease-specific survival), and PFS (progress-free survival). The five types of tumor cases were split by setting “autoselect best cutoff” and the hazard ratio (HR), log-rank *P-value* and 95% confidence intervals were computed, as well as the Kaplan-Meier survival plots were generated. We also set up other grouping factors to obtain subgroup analysis data of CARM1 mRNA expression and prognosis.

### Gene alteration and survival analysis

Analysis of gene alteration of CARM1 in pan-cancer was conducted by querying the Cbioportal tool (http://www.cbioportal.org/) [38]. The “TCGA Pan Cancer Atlas Studies” module was selected to get the genetic alteration characteristics of CARM1. The “comparison” module was also used to obtain survival prognosis data of cancer cases from TCGA (with or without CARM1 gene alteration), and Kaplan Meier plots with log-rank *P-value* were generated.

### Tumor immune microenvironment analysis

To explore the correlation between gene expression and immunotherapeutic response biomarkers TMB and MSI, sangerbox tool was used with the query of “CARM1” (http://sangerbox.com/Tool). TMB was calculated as the total mutation incidences per million base pair, and MSI was counted by the number of insertion or deletion events that occurred in repeating sequences of genes. Spearman method was used and the *P-value* as well as partial correlation value were obtained. We also conducted a co-expression analysis between CARM1 and mismatch repair genes (MMRs), methyltransferases as well as acknowledged immune checkpoints genes respectively. The images were modified using the software Adobe Illustrator.

To predict the presence of infiltration stromal or immune cells in pan-cancer tissues, R-package “estimate” and “limma” were used to calculate the scores of immune and stromal cells. As a database derived-web tool for immune cell infiltration calculation, TIMER provides the infiltration scores of many common types of immune cells [39]. Herein, we downloaded the infiltration data from it and used to test the correlation with CARM1 expression. The TIMER, EPIC, MCPCOUNTER, XCELL, CIBERSORT, CIBERSORT-ABS and QUANTISEQ algorithms were applied for immune cells infiltration calculation.

### CARM1-associated genes enrichment analysis

On the STRING website (https://string-db.org/), we firstly input the gene name and the organism “*Homo sapiens*”, then set the parameters as Network type [full network], meaning of network edges [evidence], active interaction sources [experiments], minimum required interaction score [medium confidence (0.400)] and max number of interactors to show [(“no more than 50 interactors” in 1st shell] and finally obtained the top 50 binding proteins network of CARM1. According to the data sets of all TCGA tumors and normal tissues, the “Similar Gene Detection” module of GEPIA2 was used to obtain the top 100 CARM1 related genes. The “Correlation Analysis” module of GEPIA2 was also used to perform a pairwise gene Pearson correlation analysis between CARM1 and the selected genes. The log2 TPM was applied for the dot plot and the *P-value* as well as the correlation coefficient (R) were indicated. After that, the “Gene_Corr” module of TIMER2 was applied to generate a heatmap containing the partial correlation Fcorand *P-value* in the purity-adjusted Spearman’s rank correlation.

Combining the two sets of data, KEGG (Kyoto Encyclopedia of genes and genomes) pathway analysis was carried out by uploading the gene lists to DAVID (https://david.ncifcrf.gov/), Database for annotation, visualization, and integrated discovery). The analysis results are visualized with “ggplot2” R software package.

## Supplementary Information

**Additional files 1: Figure S1.** Structural characteristics and evolutionary relationship of CARM1 protein in different species.

**Additional files 2: Figure S2.** The RNA and protein expression profile of CARM1 in various cancers and blood cells.

**Additional files 3: Figure S3.** Tumors with no significant correlation between CARM1 expression and pathological stages.

**Additional files 4: Table S1.** Subgroup analysis on the correlation of CARM1 expression and prognosis of gastric cancer cases.

**Additional files 5: Table S2.** Subgroup analysis on the correlation of CARM1 expression and prognosis of liver cancer cases.

**Additional files 6: Table S3.** Subgroup analysis on the correlation of CARM1 expression and prognosis of ovarian cancer cases.

**Additional files 7: Table S4.** Subgroup analysis on the correlation of CARM1 expression and prognosis of lung cancer cases.

**Additional files 8: Figure S4.** Tumor prognosis information from Kaplan Meier plotter.

**Additional files 9: Figure S5.** Correlation between CARM1 and some known immune checkpoints mRNA expression in various cancers from TCGA.

## Data Availability

The authors certify that all the original data in this research could be obtained from a public database. The expression data of CARM1 are available in TIMER2 (http://timer.cistrome.org/), GEPIA2 (http://gepia2.cancer-pku.cn/) and UALCAN (http://ualcan.path.uab.edu/analysis-prot.html), which sources were from TCGA (https://portal.gdc.cancer.gov/) and CPTAC. Immunohistochemistry images were downloaded from HPA (https://www.proteinatlas.org/humanproteome/pathology). The survival prognosis data could be found in GEPIA2 and verified by Kaplan-Meier Plotter (https://kmplot.com/analysis/). Gene alteration data of CARM1 can be found by querying the Cbioportal tool (http://www.cbioportal.org/). Immune related data were downloaded from TIMER2 and sangerbox (http://sangerbox.com/Tool), which sources were from UCSC (https://xena.ucsc.edu/).

## Abbreviations

PRMT: Protein arginine methyltransferase
CARM1: Coactivator associated arginine methyltransferase 1
PH-like: Pleckstrin homology-like
SAM: S-adenosyl methionine
LSD1: Lysine demethylase 1
TCGA: The Cancer Genome Atlas
HPA: Human Protein Altas
TMB: Tumor mutation burden
MSI: Microsatellite instability
TIMER2: Tumor immune estimation resource, version 2
GEPIA: Gene Expression Profiling Interactive Analysis, version 2
GTEx: Genotype-Tissue expression
CPTAC: Clinical proteome tumor analysis alliance
OS: Overall survival
DFS: Disease-free survival
DMFS: Distant metastasis-free survival
RFS: Relapse-free survival
PPS: Post-progression survival
FP: First progression
DSS: Disease-specific survival
PFS: Progress-free survival
HR: Hazard ratio
MMRs: Mismatch repair genes
DAVID: Database for annotation, visualization, and integrated discovery
FANTOM5: Function annotation of the mammalian genome 5
BLCA: Bladder urothelial carcinoma
BRCA: Breast invasive carcinoma
CHOL: Cholangio carcinoma
COAD: Colon adenocarcinoma
ESCA: Esophageal carcinoma
HNSC: Head and neck squamous cell carcinoma
LIHC: Liver hepatocellular carcinoma
LUAD: Lung adenocarcinoma
LUSC: Lung squamous cell carcinoma
READ: Rectum adenocarcinoma
STAD: Stomach adenocarcinoma
THCA: Thyroid carcinoma
UCEC: Uterine corpus endometrial carcinoma
PCPG: Pheochromocytoma and paraganglioma
PRAD: Prostate adenocarcinoma
KICH: Kidney Chromophobe
KIRC: Kidney renal clear cell carcinoma
DLBC: Diffuse large B-cell lymphoma
THYM: Thymoma
IHC: Immunohistochemistry
ACC: Adrenocortical carcinoma
UCS: Uterine carcinosarcoma
KIRP: Kidney renal papillary cell carcinoma
LGG: Brain lower Grade Glioma
MESO: Mesothelioma
SKCM: Skin cutaneous melanoma
UVM: Uveal melanoma
ER: Eestrogen receptor
CESC: Cervical squamous cell carcinoma and endocervical adenocarcinoma
AML: Acute myeloid leukemia
TME: Tumor microenvironment
PAAD: Pancreatic adenocarcinoma
SARC: Sarcoma
TGCT: Testicular germ cell tumors
