# Supplementary material for: Systematic pan-cancer landscape identifies CARM1 as a potential prognostic and immunological biomarker
Source: BMC Genom Data. 2022 Jan 16;23:7. doi: 10.1186/s12863-021-01022-w (PMC8761291; doi:10.1186/s12863-021-01022-w)
Supplement: Supplementary file 1 — Additional files 1: Figure S1. Structural characteristics and evolutionary relationship of CARM1 protein in different species. [file 12863_2021_1022_MOESM1_ESM.pdf]

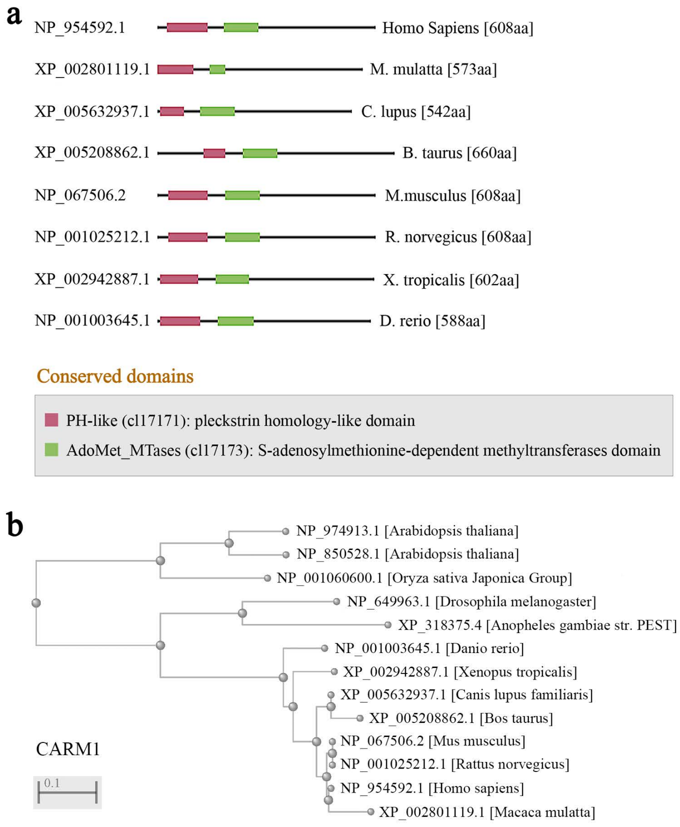

Fig. S1. Structural characteristics and evolutionary relationship of CARM1 protein in different species. (a) Conserved domains of CARM1 protein among different species. (b) The COBALT tool of NCBI was used to generate the phylogenetic tree of CARM1. The distance scale used to measure the length of evolutionary branches (also called genetic variability) is shown in the lower left corner of the image.
