# Supplementary material for: Systematic pan-cancer landscape identifies CARM1 as a potential prognostic and immunological biomarker
Source: BMC Genom Data. 2022 Jan 16;23:7. doi: 10.1186/s12863-021-01022-w (PMC8761291; doi:10.1186/s12863-021-01022-w)
Supplement: Supplementary file 2 — Additional files 2: Figure S2. The RNA and protein expression profile of CARM1 in various cancers and blood cells. [file 12863_2021_1022_MOESM2_ESM.pdf]

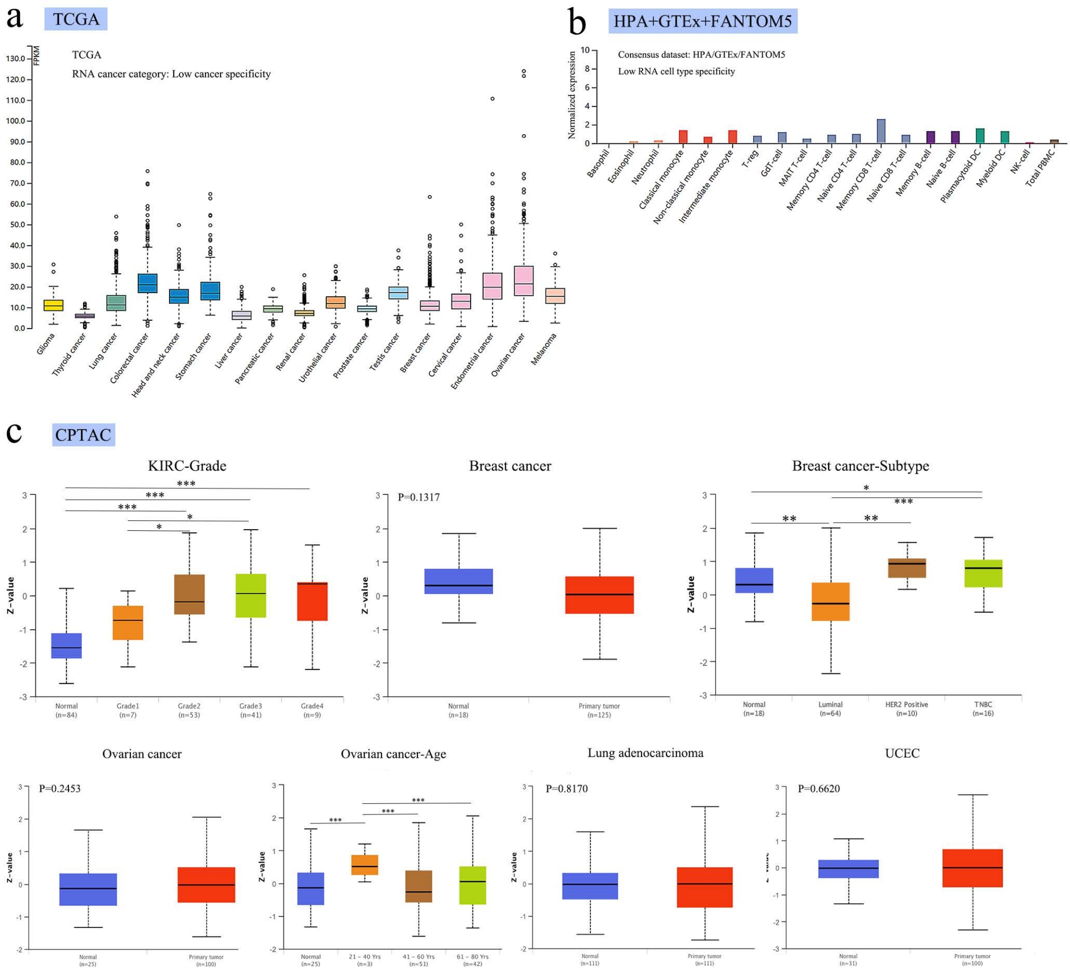

Fig. S2. The RNA and protein expression profile of CARM1 in various cancers and blood cells. (a) CARM1 RNA expression in tumor cell lines. (b) CARM1 RNA expression in different blood cells. (c) Protein expression data from CPTAC for KIRC, breast cancer, ovarian cancer, lung adenocarcinoma and UCEC, as well as clinical phenotypes of these tumors, are shown above.
