# Supplementary material for: Systematic pan-cancer landscape identifies CARM1 as a potential prognostic and immunological biomarker
Source: BMC Genom Data. 2022 Jan 16;23:7. doi: 10.1186/s12863-021-01022-w (PMC8761291; doi:10.1186/s12863-021-01022-w)
Supplement: Supplementary file 3 — Additional files 3: Figure S3. Tumors with no significant correlation between CARM1 expression and pathological stages. [file 12863_2021_1022_MOESM3_ESM.pdf]

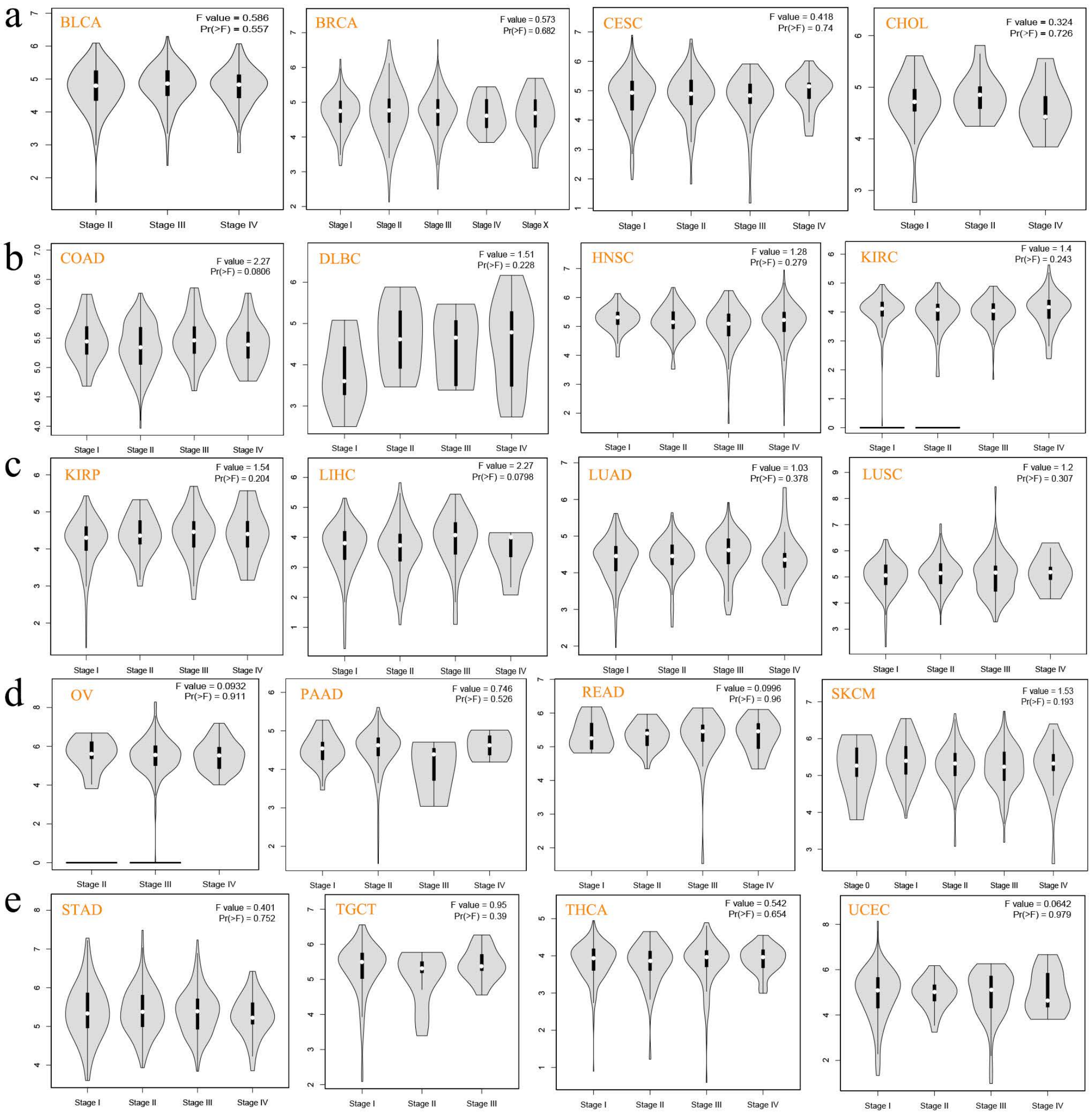

Fig. S3. Expression levels of the CARM1 gene by different pathological stages of (a) BLCA, BRCA, CESC, CHOL; (b) COAD, DLBC, HNSC, KIRC; (c) KIRP, LIHC, LUAD, LUSC; (d) OV, PAAD, READ, SKCM; (e) STAD, TGCT, THCA, UCEC.
