# Supplementary material for: Systematic pan-cancer landscape identifies CARM1 as a potential prognostic and immunological biomarker
Source: BMC Genom Data. 2022 Jan 16;23:7. doi: 10.1186/s12863-021-01022-w (PMC8761291; doi:10.1186/s12863-021-01022-w)
Supplement: Supplementary file 8 — Additional files 8: Figure S4. Tumor prognosis information from Kaplan Meier plotter. [file 12863_2021_1022_MOESM8_ESM.pdf]

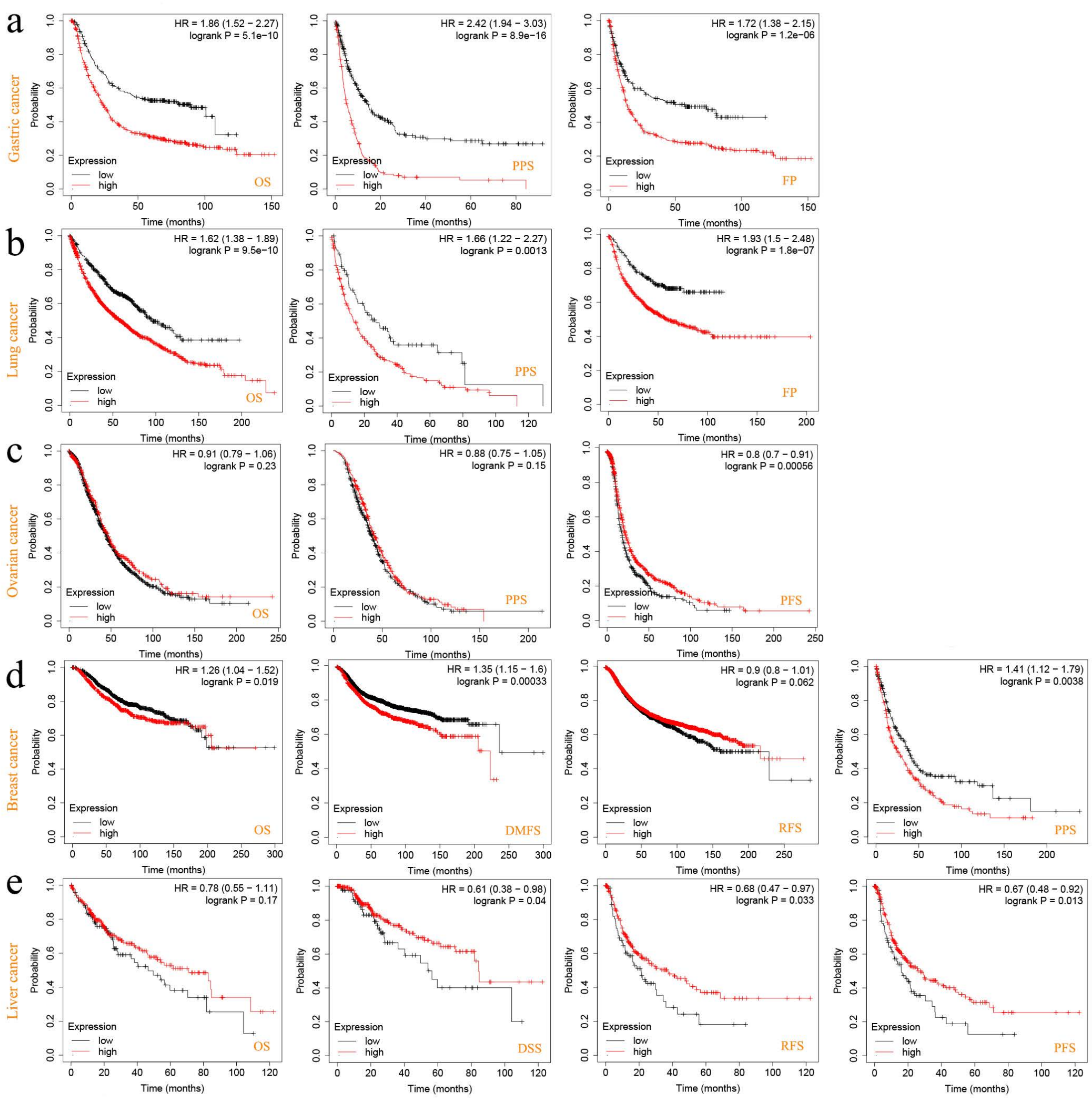

Fig. S4. **Tumor prognosis information from Kaplan Meier plotter.** Using the Kaplan Meier plotter, survival analysis including OS, DMFS, PPS, FP, DSS and PFS on the relationship between CARM1 expression and tumor prognosis was performed. Information about the prognosis of gastric cancer (a), lung cancer (b), ovarian cancer (c), breast cancer (d) and liver cancer (e) has been shown in the figure.
