# Supplementary material for: Systematic pan-cancer landscape identifies CARM1 as a potential prognostic and immunological biomarker
Source: BMC Genom Data. 2022 Jan 16;23:7. doi: 10.1186/s12863-021-01022-w (PMC8761291; doi:10.1186/s12863-021-01022-w)
Supplement: Supplementary file 9 — Additional files 9: Figure S5. Correlation between CARM1 and some known immune checkpoints mRNA expression in various cancers from TCGA. [file 12863_2021_1022_MOESM9_ESM.pdf]

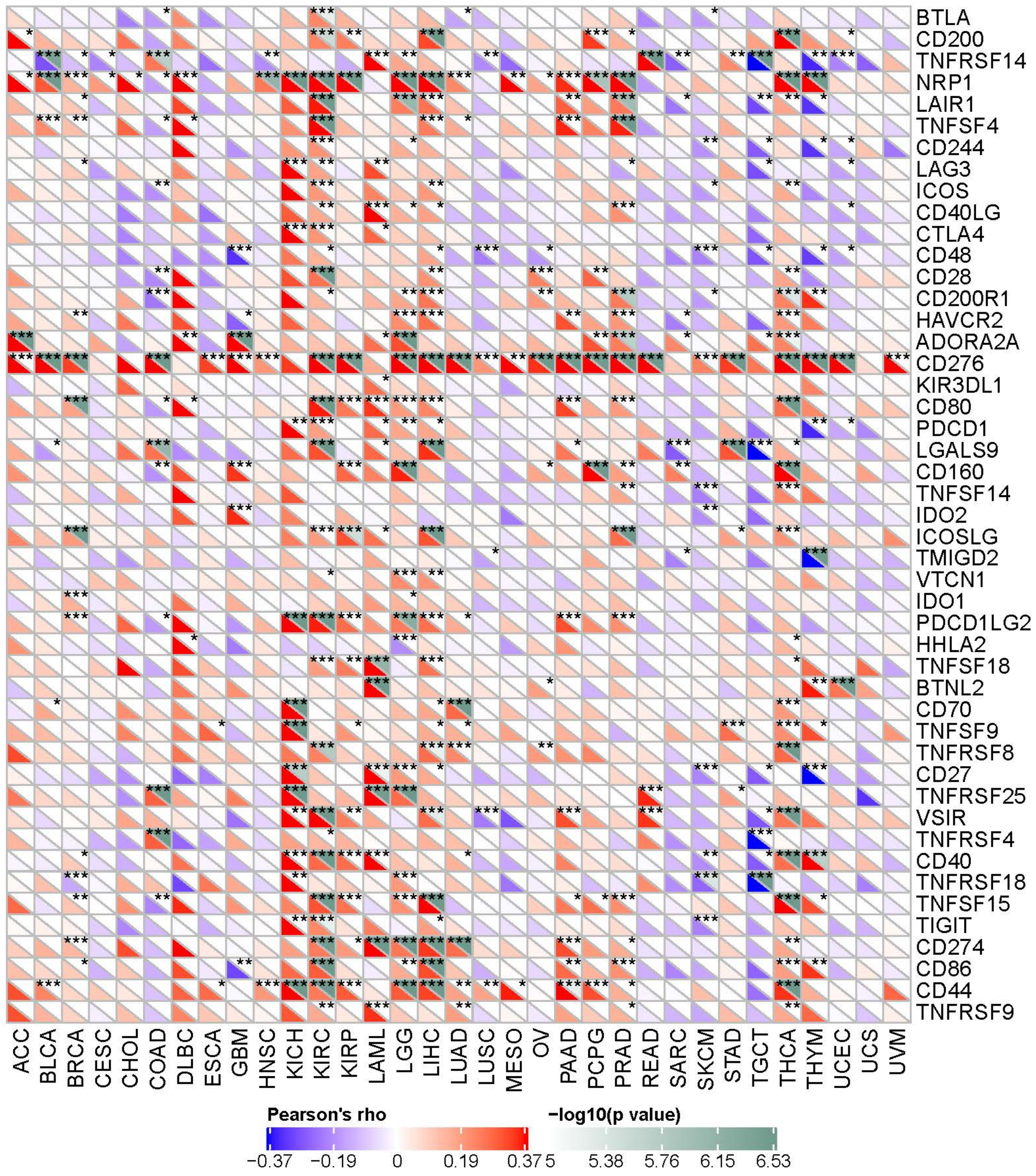

Fig. S5. Correlation between CARM1 and some known immune checkpoints mRNA expression in various cancers from TCGA. The lower triangle in each block represents the coefficient calculated by Pearson's correlation test, and the upper triangle represents log10 transformed *P-value*. * *P<0.05,* ** *P<0.01,* *** *P<0.001.*
